# Facial Fracture After Motorcycle Collision

**Published:** 2014-09-30

**Authors:** Alexis L. Parcells, Janet Yueh, Frank Ciminello, Mark Granick

**Affiliations:** Department of Surgery, Division of Plastic Surgery, Rutgers New Jersey Medical School, Newark, NJ

**Keywords:** nasoorbitoethmoid fracture, NOE fracture, facial fracture, panfacial fracture, motorcycle collision

**Figure:**
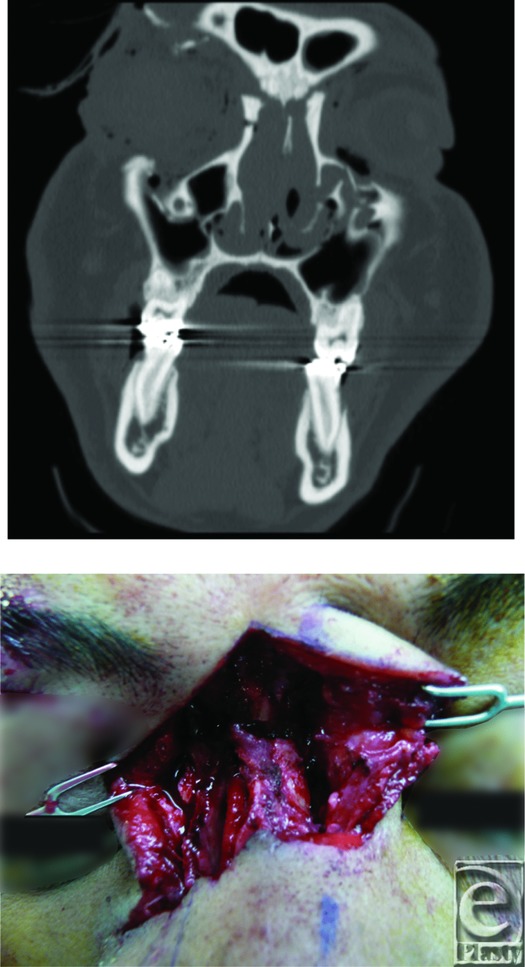


## DESCRIPTION

A 34-year-old African American man presents to the emergency department after an accident with a high-speed motorcycle. The patient was reportedly wearing a helmet. He lost consciousness and complained of pain around his right eye.

## QUESTIONS

1. **Identify fractures in the patient's CT scan.**
2. **What signs and symptoms are important in forming an accurate diagnosis?**
3. **Describe the classification system for this type of fracture.**
4. **Describe how to treat the fracture pattern in this patient.**

## DISCUSSION

The patient suffered fractures to his nasal bone, right superior and inferior orbital rims, bilateral maxillary sinuses, and lamina papyracea, consistent with a nasoorbioethmoid (NOE) fracture pattern.

Panfacial fractures may result from penetrating injury (gunshot wound, stabbing knife) or blunt trauma (motor vehicle collision). The severity of a facial injury is determined by the mass, density, and shape of the colliding object. Once the patient was medically stabilized and cervical spinal injury was ruled out, a complete examination of the face revealed a complex laceration of the nasal complex, telecanthus, and a ruptured right globe with absent light perception on the right side. Computed tomographic (CT) scan of the head confirmed multiple facial fractures within the nasoorbitoethmoid complex (NOE).

The NOE complex represents an intricate anatomical confluence of frontal, nasal, ethmoid, and orbital bones with their associated sinus tracts. Traumatic force that disrupts the primary buttresses of the NOE complex will comminute the complex and result in telecanthus, enophthalmos, diplopia, and possible cerebrospinal fistula. The NOE classification system described by Markowitz et al (1991) is based on the anatomic relation between the medial canthal tendon and the fracture. Type I fractures represent a single noncomminuted central fragment without canthal disruption, type II fractures are comminuted but the medical canthal tendon remains firmly attached to bone, and type III fractures result in severe central fragment comminution with complete disruption of medial canthal tendon insertion. The patient described earlier demonstrated complete detachment and isolation of the right medial canthus with free floating left canthus attached to bone.

Once diagnosed, the goal of treatment is surgical correction for fractures that demonstrate instability on manual manipulation or by displacement on CT scan. While the surgical approach is determined by the type of NOE fracture pattern, the focus of all NOE correction is proper insertion of the medial canthal tendon onto the bony central fragment in order to maintain the normal intercanthal distance and outward appearance of the midface. In the patient, transnasal wiring was used to position comminuted bone fragments to the medial canthal tendons and the canthi were fixated posteriorly and superiorly together. The supraorbital rim was corrected with a titanium plate, and a calvarium bone graft was harvested and placed into the lower portion of the frontal bone above the nasal root. Surgery was tolerated without complications. Enucleation of the ruptured right globe was performed on a later date.

NOE fractures are complex and encompass frontal, nasal, ethmoid, and orbital anatomy. Diagnosis is obtained through CT scan, and the goal of treatment is reduction of fracture segments and reinsertion of the medial canthal tendon to avoid telecanthus.
